# Clinical leadership and prevention in practice: is a needs led preventive approach to the delivery of care to improve quality, outcomes and value in primary dental care practice a realistic concept?

**DOI:** 10.1186/1472-6831-15-S1-S2

**Published:** 2015-09-15

**Authors:** Colette Bridgman, Michael G McGrady

**Affiliations:** 1 Public Health England, 4th Floor, 3 Piccadilly Place, London Road, Manchester, M1 3BN, England, UK; 2 University of Manchester, School of Dentistry, Higher Cambridge St, Manchester M13 9PL, England, UK

## Abstract

**Background:**

There is a need to improve access to, and the quality of, service delivery in NHS primary dental care. Building public health thinking and leadership capacity in clinicians from primary care teams was seen as an underpinning component to achieving this goal. Clinical teams contributed to service redesign concepts and were contractually supported to embrace a preventive approach.

**Methods:**

Improvement in quality and preventive focus of dental practice care delivery was explored through determining the impact of several projects, to share how evidence, skill mix and clinical leadership could be utilised in design, implementation and measurement of care outcomes in general dental practice in order to champion and advocate change, during a period of substantial change within the NHS system.

The projects were:

1. A needs-led, evidence informed preventive care pathway approach to primary dental care delivery with a focus on quality and outcomes.

2. Building clinical leadership to influence and advocate for improved quality of care; and spread of learning through local professional networks. This comprised two separate projects: improved access for very young children called “Baby Teeth DO Matter” and the production of a clinically led, evidence-based guidance for periodontyal treatment in primary care called “Healthy Gums DO Matter”.

**Results:**

What worked and what hindered progress, is described. The projects developed understanding of how working with ‘local majorities’ of clinicians influenced, adoption and spread of learning, and the impact in prompting wider policy and contract reform in England.

**Conclusions:**

The projects identified issues that required change to meet population need. Clinicians were allowed to innovate in an evironment working together with commissioners, patients and public health colleagues. Communication and the development of clinical leadership led to the development of an infrastructure to define care pathways and decision points in the patient's journey.

## Background

Implementation of efforts to improve innovation and quality can seem like abstract concepts to busy clinicians [1,2]. Implementing change can often be challenging for clinicians making decisions on a variety of theoretical models, while maintaining high standards of clinical practice [3]. Perception of clinical leadership beyond the remit of their own practice is a recent concept for many dental clinicians [4]. Public health clinicians understand the wider meaning of innovation and quality and are well placed to stimulate ‘public health thinking’ and awareness of the need for change within clinical teams. They understand factors that facilitate and hinder progress in commissioning and contracting systems, to challenge the status quo, and can work objectively with commissioners and clinicians to test new principles and approaches to the delivery and contracting of care to improve quality, value and outcomes to benefit patients.

By working in partnership with commissioners, empowering clinical leaders to shape change and spread adoption with peers, progress can be achieved and policy influenced. This paper will describe dental public health initiatives, and the impact they have had, through several case studies. These projects facilitated in the North of England have stimulated clinicians to become local leaders, they have influenced institutions, organisations and policy makers and most importantly have had an impact on the quality, value and outcomes of care delivery to benefit patients. The three projects spanned a period of substantial change in the NHS through a period of transition. Clinicians are now encouraged to develop leadership skills and be involved in the process of commissioning decisions and service redesign.

The NHS is the publicly funded healthcare system in the UK and the largest single-payer healthcare system in the world. Primarily funded through general taxation, it provides universal healthcare with most services free at the point of access. Since its inception in 1948 dentistry has been part of the offer. Means tested patient charges were later introduced; and are currently levied in primary dental care but are considerably less than equivalent charges in private dental services.

The majority of dental care in the UK is provided in primary care by dental independent contractors who offer services to patients in return for payment. Their relationship is governed by a contract. The contract was based on an item of service (IOS) for payment (often later described as a ‘drill and fill’ treadmill), apart from a small element of capitation introduced in 1990 the contract and system was largely unchanged until contract reform in 2006.

Oral health needs in England had changed markedly from 1948, confirmed by national surveys of adult and child oral health performed around the time of the development of the 2006 NHS dental contract [5,6], making the change from a contract based on delivery of treatment items to a contract incentivising a preventive approach to care a priority. The 2006 contract reform process introduced more explicit controls and a steady (and capped) monthly income to practices, with three bands of treatment activity and patient charge underpinning the contract and system.

The contract is based on an annual contract value and activity measured by units of dental activity (UDAs) relating to three bands of care. UDAs were related to activity in the fee for item system so cost and volume of treatment activity remains the currency. No needs assessment, access or outcome measures were introduced formally to performance manage service providers; so the completion of a true commissioning cycle is almost impossible.

As might have been predicted (and intended) treatment activity has fallen markedly in NHS primary dental care delivery since 2006, associated with changes to primary care dentists’ practicing profiles [7]. However, as there is no explicit funding for preventive intervention and no measurement of individual patient need/risk or outcome of care, the contract requires further reform; and that is now under consideration by the Department of Health (DH) policy makers.

Patients with higher needs are seemingly less welcome and there is evidence that ‘well patients’ receive a disproportionate share of the resource - available clinical time - for over frequent check-up and dental prophylaxis. It is against this background that these case studies were initiated. There is general agreement that a treatment activity, contract and system, is no longer compatible and a shift to a preventive approach is required [8].

The Health and Social Care Act 2012 came into effect in England in April 2013 [9]. Under the new system a new commissioning board called NHS England now oversees the commissioning of all NHS dentistry in England. Commissioning guidance was published: “Securing Excellence in Commissioning NHS Dental Services” in February 2013 [10].

The DH and NHS England are currently exploring further contact reform and are piloting a preventive care pathway approach (this is based on the redesign model from case study 1) in almost 100 NHS dental practices in England to improve NHS dental care. As the single commissioner for dentistry NHS England is responsible for making sure all services, including dentistry, focus on improving health outcomes not just the delivery of items of treatment activity.

Commissioning can be summarized as securing good value services, to meet identified need and improve outcomes, within available resources; working in collaboration with clinicians, patients and/or the public. It translates aspirations and need, by specifying and procuring services for the local population, into services for users which deliver a preventive approach and the best possible health and wellbeing outcomes within resources [11].

In the context of health-care, quality should have a universal meaning - the right competence and skill level of clinician, doing the right thing well, at the right time, for the right reasons to obtain the best achievable health outcomes and value for money. Although there is probably little disagreement with the definition, there is less agreement about what is effective commissioning and how quality and value should be measured and can be incentivised.

Commissioning is a dynamic cyclical process with four distinct areas of work that support the approach; the first of which is analysis and needs assessment. Each section of the cycle requires contracting processes. Much of what is often referred to as commissioning in dentistry in England has is in fact been contracting.

If population and individual patient need/risk is not measured and reassessed at intervals, the extent to which services have contributed to improved outcomes cannot be captured or monitored.

The second part of the commissioning cycle is planning, drawing up a strategic prospectus to meet identified need and serve demand; it will often include service redesign options and the development of outcome measures. Contracting processes that underpin this phase are service specification, drawing up a contract and/or service level agreement that includes outcome measurement.

The third part of the cycle is managing provider relationships and stimulating the market by building clinical capacity to meet new service design requirements. Contracting supports this part of the cycle by procurement, securing providers and managing contracts.

Finally, the cycle is completed by the review section. This includes a review of the strategic plan and performance of services, to meet need and improve outcomes as intended. The contracting process which supports this phase is about monitoring and is based on collation and reporting of individual provider outcomes and performance [11].

It all begins again with a refresh of the first phase. This starts as before with analyses, understanding need, knowing available resources and so on. At this stage good commissioners, having used resources effectively, will have an opportunity to reinvest savings or utilise resources from ineffective services by decommissioning.

That is the theory. In practice in NHS dentistry the commissioning cycle can rarely be conducted in this way or to this degree of rigour. This is due in part to a lack of, robust evidence of what works in treatment service delivery, individual needs assessment and monitoring of outcomes. A contracting approach that relies on volume, cost and activity pervades. Established clinical culture and a lack of effective commissioning levers in the system also contribute.

Seeking ‘value for money’ within NHS commissioning is often perceived, and has often been, blunt cost reduction; that is not commissioning in its true sense. If value is defined, as outcomes relative to costs, it should also encompass efficiency and quality. Therefore achieving value should depend on results for patients, not just inputs. Value should be measured in terms of allocative and technical value, and by the outcomes achieved, not just on the volume of services delivered. The costs should include the full cycle of a patient's journey and not just the cost of individual services or programmes. To reduce cost, the best approach is often to spend more on some services to reduce the need for others. Decisions that are just cost reduction, without regard to the outcomes, leads to ‘false’ short term savings and potentially it limits effective care delivery. Measuring, reporting, and comparing outcomes is needed and would assist in improving performance, allow peer review pressure to be transparent and support making good choices about where to reduce costs (decommission where care is sub-standard). This is true for many service areas and has particular resonance for dentistry. It was this concept that was the catalyst for the work described in the first case study: Primary Dental Care Service Redesign in three dental practics in Oldham and Salford, North West England.

The second and third case study projects took place after the NHS reforms resulting from the Health and Social Care Act 2012, in the context of the establishment of NHS England as commissioner and the development of clinical leadership: Building clinical leadership to influence and advocate for improved quality of care; and spread of learning through local professional networks. Two work streams developed from this: “Baby Teeth DO Matter” designed to improve access to primary care for very young children; and “Healthy Gums DO Matter” consisting of a clinically led evidence-based guidance for the management of periodontal disease in Primary Care.

## Methods

A common theme ran through the methodology for each of the case studies, namely the development of clinical leadership within the primary care teams involved in each of the projects. The extent of the leadership input from clinicians increased through the time period of the projects to reflect the changing paradigm of the NHS in England.

### Case study 1: Primary dental care service redesign

In 2007, within the regulatory framework, the use of a care pathway approach in general dental practice was designed and evaluated. This was based on a structured and consistent assessment of clinical disease and risk via an Oral Health Needs Assessemnt (OHNA) and the monitoring of adherence to and the impact of an evidenced informed care pathway. The methodology and background for this work has been previously reported in the literature [12,13].

In summary, three dental practices were selected to develop and test the new system. One was a newly commissioned practice located in a high deprivation area of Oldham, with a high proportion of ethnic minority groups. The other two practices were established practices located in Salford, selected because of a willingness to work with commissioners and the dental public health team in an innovative way to improve service delivery. Both practices were located in disadvantaged areas and recognised difficulties in moving towards a preventive approach within the 2006 NHS dental contract. They were willing to collaborate and work with commissioners to redesign the primary dental care offer, working innovatively to develop a preventive, needs led approach and lead change in their practice teams.

Through the establishment of a steering group and a series of workshops, clinical dental teams exploited integrated use of skill mix [14,15] to adhere to the preventive care pathway within existing resources. The model included timely access to care for new patients (routine and urgent) with an emphasis on patient self-care responsibility.

**Figure 1:**
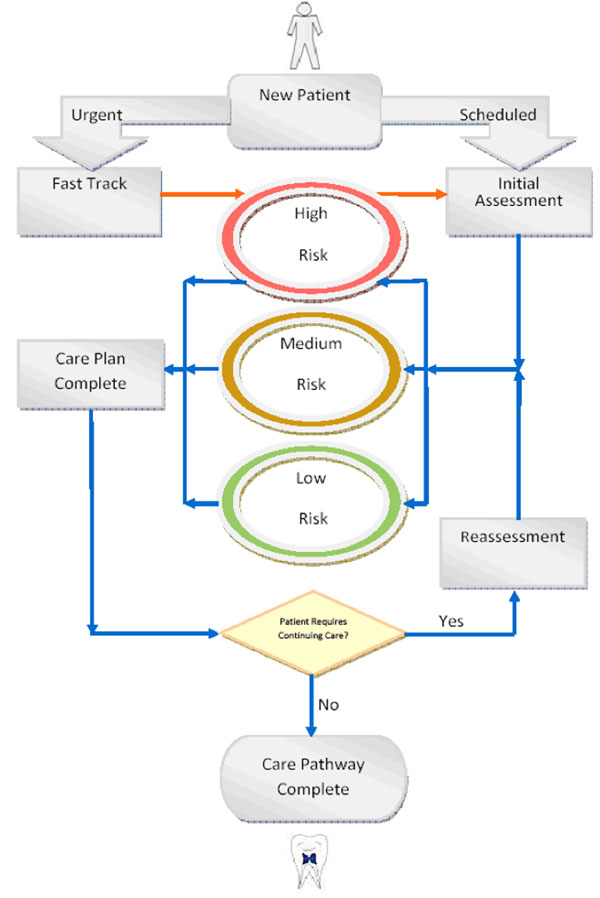
Illustration of care pathway for patients.

The new system included the use of an OHNA, which included information on medical history, social history, previous disease, diet, oral hygine practices and clinical examination. Ethical and NHS Research and Development approval was obtained in order to carry out an evaluation of the pilot. Based on consistent OHNA information patients were assigned to diagnostic and pathway groups for caries and periodontal disease using a high, moderate and low, traffic-light coding system that described need and risk. This is illustrated in Figure 1. Preventive interventions and treatment plans were then governed by guidelines, relevant for the age-group and condition, within a pathway; to prompt evidence informed advice and action and to support clinical decision making within agreed care protocols and directed recall periods.

The implementation of the care pathways was adaptive and responded to collection of data from patient outcomes such as patient satisfaction, financial impact and clinical outcomes.

### Case study 2: “Baby teeth DO matter"

The second case study took place following the Health and Social Care Act (2012). During this time it was known PCTs would be abolished by the 1^st^ April 2013 and NHS England (formally the NHS Commissioning Board) would assume commissioning responsibilities for NHS dental services. The key principles of: “Securing Excellence in Commissioning NHS Dental Services” [10] was to re-orientate dental services so that they become focused on quality, outcomes and meet identified patient and population need.

NHS England responded by establishing local professional networks for dentistry (LPNs) to ensure that clinicians could influence dental commissioning. In Greater Manchester a “shadow” LPN was established to test and understand what added value could be gained by putting local clinicians at the heart of commissioning decisions. The benefit of involving dental clinicians in this way was tested in two LPN initiatives 1. “Baby Teeth DO Matter” (BTDM) in 2013 and 2. “Healthy Gums DO Matter” (HGDM) in 2014. The establishment of the LDN enabled commissioners and the dental public health team to build on the learning of the first case study and broaden the scope of clinical leadership beyond the three initial practices who initially engaged, to include clinicians and primary care teams across a larger geographical footprint - the NHS England area team for Greater Manchester encompassed 477 practices formerly commisioned by ten former PCTs.

BTDM was initiated to address identified poor OH and access to primary dental care for very young children in Greater Manchester. Public Health data confirmed that decay was impacting on many children from a very young age and many of those most in need were not receiving access to preventive services; symptoms were prompting access to dental care; often for the extraction of deciduous teeth under a general anaesthetic after a child experienced pain and sleepless nights.

General dental practitioners worked within a “shadow” LPN to assist in the development of a practice based initiative to encourage identification of very young children (in the community close to their practice) who were not benefiting from access to preventive dental service and intervention. Practitioners recognised that they were seeing many young children too late and that most had missed out on available preventive interventions and advice. One of the practitioners engaged in the programme suggested the phrase “Baby Teeth *DO* Matter” as a project title as he had so often heard parents (told that their child needed extractions) say ‘well it's only the baby teeth so they don't matter’. The project clinicians worked to design an intervention that would incentivise Greater Manchester General Dental Practitioners (GDPs) to:

• Seek out very young children (under five years old), in the community of their practice location, who had not attended for routine dental care

• Make every contact count, with advice to the parents by delivering simple evidence-based oral health messages based on Delivering Better Oral Health (DBOH) and encouraging future regular attendance.

A key aspect of the programme was to ensure local GDPs were involved at every stage of the planning and running of the programme. A small group of selected clinicians developed the programme and a second tier of local clinicians located in each of the localities of the former PCTs was engaged to spread learning within their local clinical networks across the footprint and population of Greater Manchester. These were called “*clinical champions*” and their role was to oversee the initiative in their locality, sign-up new practices, help participating practices to become community-facing and liaise with Oral Health Improvement Teams. Analogous to the developments in medicine, “*clinical champions*” the clinicians on the shadow LPN were encouraged to become “*clinical leaders*”. A paediatric sub-group was also formed to enable the “shadow” LPN practitioners to work with paediatric primary and secondary care specialist clinicians to develop clinical care pathways for young children.

This initiative subsequently became Phase II of the “Baby Teeth *DO* Matter” programme and resulted in a booklet entitled “*Good Practice Guidelines for the management of 3-4 year old children in primary care*” published for practices in Greater Manchester. The booklet provided advice and guidance for busy NHS practices on proactive prevention and local treatment to reduce referrals for care under General Anaesthesia.

### Case study 3: “Healthy gums DO matter"

The Greater Manchester LPN, having gained experience and learning from the BTDM work, embarked on a second initiative in early 2014. A core sub group was established to plan and implement this second project. From previous experience this had to include dental clinicians and teams, commissioners, patients and dental public health; working together.

The aim was to produce a periodontal resource toolkit for primary dental care teams in Greater Manchester. The intention was to compliment the evidence informed guidance on prevention with evidence informed guidance on periodontal care and treatment pathways to support Greater Manchester primary care teams to improve outcomes for patients experiencing periodontal disease.

The initiative was led by a general dental practitioner; who realised his leadership potential in the first service redesign project (case study 1) and in his work with BTDM. He was determined that the periodontal toolkit will distil the evidence and specialist guidance available into workable pathways for NHS primary dental care practice in Greater Manchester.

The care pathways have been developed according to clinical need and the project will therefore describe need and outcomes of care for patients attending NHS primary dental care in Greater Manchester. Everyone involved understands that this project aims to use the existing investment more effectively by facilitating teams to appropriately manage periodontal care in NHS dental practice. Commissioners are therefore integral to the work and are exploring mechanisms to support change within contract regulations by adapting a blended approach as tested in BTDM. Progress is being made because primary dental care teams are being given the tools, knowledge and confidence to deliver evidence informed best practice for periodontal disease with patients understanding their responsibility in self-care.

The lead practitioners recognise the gap between how care is being delivered in NHS practice and the recommended guidance. The LPN acknowledges the gap and this initiative aims to bridge that disconnect. The move to best practice will be an on-going journey and the acceptance and uptake of the toolkit by GDPs is crucial. The task has a number of phases. First has been the development and agreement of the toolkit, next it is being tested, with a service level agreement to ensure appropriate remuneration, in 10 practices.

The task group are exploiting the understanding that the single largest impact on outcomes is the patient's oral hygiene and self-care. Initial therapy in the pathway is therefore effective oral hygiene instruction; this can stabilise two thirds of diseased sites. During the course of the pathway the management moves from the patient level to a ‘mouth level’ analysis of risk factors such as plaque control to 'tooth level’ risk factor management such as tooth anatomy and finally to 'site level’ risk factor management such as bleeding on probing.

One challenge has been to try and recognise at what stage in the pathway a patient should move to more extensive periodontal therapy. If they are struggling to maintain adequate oral hygiene and plaque control their ‘need’ is to be assisted to improve that. The adequate level of plaque control has been set at 30% plaque score and 20% bleeding score, based on the use of modified abbreviated indices on Ramfjord teeth for periodontal assessment [16], before patients progress to advanced periodontal therapy, although the pathway allows clinical discretion to comensate for patient modifying factors, such as cognative impairment.

A patient agreement has been produced, not to pass all responsibility to the patient but rather it is designed to be used to encourage and educate the patient as to their self-care responsibility. It is a collaborative agreement also outlining the responsibilities of the dental team.

The clinical manual (and associated summary document), developed by the HGDM team was launched at an LPN event in November 2014 to all dental practices in Greater Manchester. The development of a clinical leadership training course to incorporate the learning and toolkits from HGDM has been completed in collaboration with a national social enterprise body - Primary Care Commissioning (PCC). This will faciltate the expanded development of clinical leadership, to enable other clinicians, wthin and beyond the scope of the HGDM project in Greater Manchester, implement and lead change within their own teams elsewhere in England to improve quality, value and outcomes in the delivery of NHS periodontal care.

## Results

### Case study 1: Primary dental care service redesign

As this was the first project initiated, there has been a greater period of time to assess the outcomes and the impact of the project. In summary, this innovative needs- led clinical care pathway model provided a structured approach to the re-orientation of dental services towards prevention in 3 NHS dental practices. The results of this case study have been previously published [13]. The Key Performance Indicators (KPIs), established during the outset, led to discusssion between the practictioners and commissioners regarding the utility and value of such indicators to capture clinical outcomes. The project identifed the key outcome measures required, to meet the objective of maintaining the Donabedian delineation: structure, process and outcome [17]. The pilot scheme demonstrated it was possible to monitor the transfer of patients bewteen the catagories outlined from the OHNA in a practical and feasible manner, through a responsive and validated mechanism.

Leadership development of dentists and training of DCPs was essential to implement this model. Considerable challenges were identified in bringing high need patients to a successful conclusion of the patient journey. This included a move away from traditional monitoring of clinical activity and towards the measurement of patient outcomes.

### What have been the benefits?

Benefits were realised across a number of dimensions. Care and increased access was delivered within resources:

• Commissioners and clinicians could now understand `need’ of a practice population

• Efficiency was evidenced by an increase in skill mix in all three practices

• Outcome indicators were developed and positive changes in the level of oral health was evidenced for many patients from assessment to review

• Increased access and quality in preventive dental service was delivered

• Increased patient experience and satisfaction and clinical team engagement was witnessed

• Most challenging patients could be identified and their needs accommodated

### Case study 2: “Baby teeth DO matter"

Care pathways were provided for young children presenting with or without symptoms. Within two months, 195 of 477 practices across Greater Manchester had signed up to Phase I of the programme (41%) and 3,453 children who had not previously accessed care had attended general dental practice. This represented a significant improvement on the numbers within this age group attending primary care dental services and developed relationships between primary dental care providers and the wider health and social care community.

Links were made with local doctors, Sure-Start Children Centres, nurseries and schools. Local GDPs worked with their Oral Health Improvement Teams and a few took the initiative to involve the local media which produced newspaper articles and local radio broadcasts. The booklet for Phase II of the programme was distributed to all of the 477 practices across Greater Manchester and launched at a Ministerial visit. An electronic training package on quality paediatric care has been developed, with Health Education England for postgraduate training; and to date over half of the practices have completed this training.

### Case Study 3: “Healthy gums DO matter"

While it is too early to evaluate the outcomes of HGDM, the early feedback from both the primary care practices involved in the pilot and the specialists located in secondary care is encouraging. Practitioners welcome guidance that is informative and evidence based, written for primary care clinicians for improved management of periodontal disease in practice. Local specialists recognised the need and benefit of practitioners being supported to manage periodontal disease in an effective and evidence based manner. Contributing to this work and improving periodontal care management in primary care will maximise the effectiveness and efficiency of the limited specialist resource available.

## Discussion

It was apparent that radical change was required in the way in which primary dental services were being delivered to move services, focused on treatment, to a more preventive model of care. This service redesign project involved collaboration, a strong team approach and clinical engagement from the outset. The project steering group was led by the Consultant in Dental Public Health (DPH) and Dental Commissioning leads in Oldham and Salford and included three dental practice teams. The essential element of the service redesign project was the equal contribution of skills and perspective from DPH, Commissioners and clinicians, and that 3-way contribution in planning, was the first learning point. The group set out to think differently about primary dental care and aimed to redesign services to capture individual need; taking account of disease experience and behavioural/medical patient risk factors to implement evidenced preventive interventions in a structured way and report outcomes.

To achieve a responsive preventive model of care that placed the patient at the centre meant that ‘early redesign thinking’ could not to be constrained by current or traditional services models and professional body regulations. These would be challenged successfully when identified as a barrier within the project. The steering group understood: to achieve improvement in oral health and dental services required that the teams worked differently and they set out to exploit flexibility in the 2006 contact to stimulate change.

The service redesign project brought four different work strands together:

• Workforce development using knowledge & competency analysis in Clinical Care [18],

• Needs assessment, the requirement to understand ‘practice population need’ for planning and monitoring of outcomes of general dental services care,

• Using evidence and effective prevention in primary care practice and

• Ensuring access to care for those patients with high dental need.

In addition to population programmes and action, there was a need to refocus dental services from repair to prevention through effective commissioning. Primary dental care, despite the 2006 NHS dentistry contract move away from `fee for item’ payment system’ was still largely `tooth and treatment focussed’. Many primary care dental teams aimed to contribute to improved oral health but too often care and preventive advice was not being delivered according to need nor was it evidence informed.

Delivering Better Oral Health (DBOH) was published by DH 2007 [19]. Evidence informed clinical preventive advice and interventions that have known efficacy needed to be tested for effectiveness, affordability and acceptance within primary dental care. Early work (in patient centred workforce planning), indicated the need for skill mix, to be embraced by dentistry and for it to be extended beyond the use of therapists and hygienists, to dental nurse development.

This project was outcome focussed and set out to measure clinical improvement from the outset. Clinical process and outcome measures were developed and tested [12,13]. The project team wanted to understand and learn which measures would be most helpful and sensitive in describing need, capturing service improvement and ultimately improved oral health. Robust evaluation was built into the project. It was intended that if the redesign approach proved effective and affordable it could be scaled up to other practices.

The context of oral health experience in the North West of England was poor in comparison to the national average [20,21]. Oldham and Salford populations had higher than average oral health needs and the impact of poor oral health in young children gave particular cause for concern.

General dental practices provide the majority of NHS dental care. The 2006 dental contract allowed dentists to move away from a fee-for-item system however dentists found it challenging to move to a preventive approach to care. The contract measures were linked to the previous finance system and as a result of this were perceived by clinicians as treatment activity targets.

The project aimed to incorporate DBOH evidence-informed preventive interventions within new commissions and improve access and quality in existing contracts. The new model of care was designed to ensure that dental treatment and preventive interventions/advice were delivered according to identified need and by the most appropriate team member.

The traditional dental team usually consists of 1 (or more) dentists, each with a dental nurse, one receptionist and possibly a part time hygienist (rarely therapist) for the practice. Most practices in Oldham and Salford are small, 1 or 2 dentists; few have more than 2 dentists. All patients saw the dentist for an examination and treatment plan followed by care provision; rarely the treatment plan involved a referral to a hygienist for scaling and preventive care. The use of a dental therapist in general dental practice was minimal. Using dental nurses ‘clinically’ to deliver preventive interventions had not been explored in general dental practice in England prior to this project.

The need to make skill mix changes evolved from ‘patient centred’ workforce development within the project. A detailed needs assessment and primary dental care task analysis was completed and this led to the radical rethink as to how primary dental care could be delivered by teams.

Regulatory body changes being introduced at the time allowed a broadening of roles undertaken by Dental Care Professionals (DCPs). Previously dental therapists, dental hygienists and dental nurses all operated according to lists of ‘permitted duties’. In 2006 the General Dental Council (GDC) suggested the activities undertaken by all DCP groups could be determined by reference to their training and competence. This removed previous restrictions to innovation in dental team working. The requirement that the dentist should see the patient first for assessment, and treatment planning, remained (Direct Access has since been permitted by the GDC in 2013).

As part of this redesign project, a course for additional training for practice dental nurses, was developed and delivered. An application was made to the GDC in August 2007 and received approval. The course was designed and established for the DCPs of the practices. The first cohort of practice dental nurses with additional skills, and competence in prevention and the application of fluoride varnish, qualified in March 2008. This ensured that all three practices had trained skill mix; required to implement the new model efficiently. The project dental nurses were the first to qualify with ‘additional skills’ and work ‘clinically’ within general dental practices in England. This was the first significant impact of the project.

The project developed 3 primary dental care teams to work in an integrated way within a needs led preventive, outcome focussed approach with adaptation to existing IT systems. All the clinicians and DCPs involved reported being more satisfied with the care they were delivering. Dental commissioners had a greater understanding of the needs of patients in these practice populations and could monitor and understand performance, in particular with regard to recall intervals. Patients in all three practices were able to access quality care in a timely manner and improvements in oral health were evidenced. The transfer of responsibility and emphasis on self-care together with a better understanding of dental disease process was well accepted by patients. A percentage of patients with higher needs continued to fail to re-attend for continuing care and the clinical teams are working to understand the barriers to care; however all patients who use services symptomatically are accommodated and invited to have full assessments and continuing care.

The learning from this project was cited in the Independent Review of Dental Services (June 2009) as an example of good practice [8]. Such was the level of interest that other areas in England expressed an intention to implement the model and care pathway. Some made formal visits to learn about the work and all were given electronic copies of the needs/risk assessment tools and clinical outcome indicators that had been developed by the original project team and clinicians. This allowed implementation of the newly developed tools and the service redesign model across a wider health care economy.

Having shared tools and elements of the pathway, so early in its development, the originators of the model, found it difficult to keep control of ensuring that a full understanding of what was necessary for successful implementation was being used in local adaptation. The use of needs assessment and the care pathway, without training and developing integrated skill mix use of DCPs or building leadership with clinical leads would be unlikely to deliver the results witnessed in Oldham and Salford. The approach was often complicated by others leading to less acceptability with clinical teams it was imposed on. This is a key learning point for those involved in initiating projects that deliver positive change. Analysis and publication of comprehensive learning, prior to wider implementation, would have ensured that all essential components necessary for successful replication were better understood. This wider and rapid adaptation resulted in numerous, but different versions of the needs/risk assessment and care pathway.

The Salford and Oldham team presented early findings in a poster presented at IADR in 2010 [22] and a description and impact of the OHNA and Care pathway approach was published in the literature [12,13].

The level of interest in the project did influence the DH to pilot a preventive care pathway in a larger number of practices in England. Investment was made by DH and workshops were held with a wider group of consultants in DPH, academics, commissioners and policy leads to develop a consistent Oral Health Assessment and Care Pathway for England. It was to be piloted in over 70 practices to test contract reform models. Dentists from the three original practices assisted, in the development and training of teams selected to take part, early in the current DH pilot programme.

A needs/risk led clinical pathway is now an accepted approach and is influencing current contract reform. It is having a positive impact on patient outcomes in the DH pilot programme. The modified IT system and the increased need to extend training of skill mix in pilot practices has probably resulted in access falling in a number of pilot practices [23]. Questions regarding the affordability of the current pilot programme could be related to this rather than the model or approach to care.

The care pathway approach has been welcomed by pilot clinical teams and patients and this is influencing a demand for wider implementation. Policy makers have confirmed that a pathway approach to care is a given and that future contract reform will support that to happen.

Following the learning from the service redesign project and the opportunities afforded by the Health and Social Care Act (2012), the role of “*clinical leadership*” in the context of the GM shadow LPN and Phase I and II of “Baby Teeth *DO* Matter” and HGDM was explored to understand the impact that empowering local clinicians had played in the development and spread of the learning. Following an analysis of the impact of each participating GDP, almost four thousand children who had not accessed dental services previously have now attended. They were each given a free toothbrush and toothpaste and two simple evidence-based messages to promote an oral healthy routine. Whilst increased access and clinical activity are only surrogate measures to determine the effectiveness of the programme, the importance of empowering local clinicians and creating “community facing” dental clinicians was demonstrated [24].

The LPN initiatives of BTDM and HGDM address four of the five key health promotion domains in the World Health Organization's Ottawa Charter (1986) [25]:

• “*re-orientating health services towards prevention*”,

• “*creating supportive environments*”,

• “*strengthening community action*” and

• “*developing personal skills*”.

The development of empowered clinicians who want to make a difference to their local community cannot be under-estimated, given their ability to create momentum for local change and influence peers. In addition, linking local clinicians to their oral health team counter-parts in the community provides a “*joined-up*” approach and ensures consistency in oral health messages being delivered at all stages of prevention, from primary to tertiary approaches.

One of the potential criticisms of both BTDH and HGDM is the use of financial incentives to drive the programme forward and encourage adoption. Using Units of Dental Activity as a currency in a blended contract approach to pay GDPs for clinical activity of this kind would appear to be sensible to shift services to a preventive approach.

Critical to the success of both programmes has been the Dental Public Health input and the “*task and finish*” resource of LPN sub groups. The former is required to provide a strategic approach to establishing and developing a clinically led LPN. It has also brought a consistent approach to the delivery of evidence-based prevention and an understanding of the levers within the NHS that can influence change.

As highlighted in “Securing Excellence in Commissioning NHS Dental Services” [10]

“*the partnership with dental public health is crucial to delivering the vision for NHS dental services*”.

Another key learning point is the importance of keeping the approach and messages simple and also ensuring good communication through the network. This will be a challenge to LPNs in the future as they seek to strategically lead their local clinicians to embrace a change in culture and delivery.

## Conclusions

In summary, this case study confirms that consultants in DPH, commissioners and clinical teams working together can develop workable innovative ideas, to overcome issues, and make changes to benefit patients and spread innovation, taking advantage of changes through evolving structures in the NHS in England and developing clinical leadership skills in primary care clinicians. Understanding population and individual patients need is an essential element in service design. Clinical leaders can innovate and are best placed to champion and advocate change with peers. The infrastructure to enable implementation as intended is necessary. Defining what should be measured, with a clear focus on outcome is needed and should be as light a touch as possible. Robust commissioning levers and correct incentives for service providers make change happen. The need for consistent and user friendly information collection tools and clinically led IT development, to support clinical adoption, and patient choice, are also key learning points from this project.

Given the lack of evidence on the effectiveness of oral health education [26], the involvement of all the relevant clinical stakeholders in delivering simple evidence-based messages to parents and children appears critical; if both access and disease severity metrics are to be improved for this younger age group [27]. It was also found important to target areas of high need to reduce health inequalities.

Through these case studies there have been factors and learning points identified. Which have helped to develop ideas, overcome issues and spread innovation.

### The key learning points are

• Identify issues that require change and define population need

• Assurance clinicians are allowed to innovate

• Always have clinicians, commissioners, patients and dental public health working together in development

• Clinical leadership is powerful to champion implementing change and to advocate with peers

• Establish infrastructure to enable communication through networks

• Define the care pathways and key decision points in the patient's journey.

• Define what should be measured; focus on outcomes to identify performance that can be used as quality standards.

• Provide ‘user friendly’ information to support clinical adoption and patient oral health literacy

• Develop levers to support

• And incentives to encourage

Essentially this describes a ‘complex adaptive system’ most systems operate in a linear fashion expecting single interventions to have a result but that is not what happens. **Complex adaptive systems** bring together all the contributors including patients [28].

LPNs need to align to overarching strategic intent but how each local network achieves progress has to be left to local decision making and local innovation because complex adaptive systems are learning systems and need to be able to act on and share action learning.

## List of abbreviations used

BASCD: British Association for the Study of Community Dentistry; BTDM: Baby Teeth DO Matter; DBOH: Delivering Better Oral Health; DH: Department of Health; DCP: Dental Care Professional; DPH: Dental Public Health; GDC: General Dental Council; GDP: General Dental Practitioner; HGDM: Healthy Gums DO Matter; IOS: Item of Service; IT: Information Technology; KPI: Key Performance Indicator; LPN: Local Professional Network; NHS: National Health Service; OHNA: Oral Health Needs Assessment; UDA: Unit of Dental Activity

## Competing interests

CB received funding from Colgate Palmolive to attend the Cape Town Conference. The authors declare they have no other competing interests.

## Authors’ contributions

CB wrote the manuscript. MGM contributed to the writing of the manuscript and additional materials. All authors read and approved the final manuscript.

## Declarations

This article has been published as part of *BMC Health Services Research* Volume 15 Supplement 1, 2015: Proceedings of Prevention in practice - making it happen. The full contents of the supplement are available online at http://www.biomedcentral.com/bmchealthservres/supplements/15/S1. Publication charges for this supplement were funded by Colgate Palmolive.
